# Leveraging 16S rRNA Microbiome Sequencing Data to Identify Bacterial Signatures for Irritable Bowel Syndrome

**DOI:** 10.3389/fcimb.2021.645951

**Published:** 2021-06-11

**Authors:** Yuxia Liu, Wenhui Li, Hongxia Yang, Xiaoying Zhang, Wenxiu Wang, Sitong Jia, Beibei Xiang, Yi Wang, Lin Miao, Han Zhang, Lin Wang, Yujing Wang, Jixiang Song, Yingjie Sun, Lijuan Chai, Xiaoxuan Tian

**Affiliations:** ^1^ State Key Laboratory of Component-Based Chinese Medicine, Tianjin University of Traditional Chinese Medicine, Tianjin, China; ^2^ School of Chinese Materia Medica, Tianjin University of Traditional Chinese Medicine, Tianjin, China; ^3^ Key Laboratory of Pharmacology of Traditional Chinese Medical Formulae, Ministry of Education, Tianjin University of Traditional Chinese Medicine, Tianjin, China; ^4^ Laboratory of Pharmacology of Traditional Chinese Medical Formulae Co-Constructed by the Province-Ministry, Tianjin University of TCM, Tianjin, China; ^5^ Tianjin Zhongxin Pharmaceutical Group Co., Ltd. Le Ren Tang Pharmaceutical Factory, Tianjin, China

**Keywords:** gut microbiome, biomarkers, 16S rRNA, machine learning algorithm, irritable bowel syndrome

## Abstract

Irritable bowel syndrome (IBS) is a chronic gastrointestinal disorder characterized by abdominal pain or discomfort. Previous studies have illustrated that the gut microbiota might play a critical role in IBS, but the conclusions of these studies, based on various methods, were almost impossible to compare, and reproducible microorganism signatures were still in question. To cope with this problem, previously published 16S rRNA gene sequencing data from 439 fecal samples, including 253 IBS samples and 186 control samples, were collected and processed with a uniform bioinformatic pipeline. Although we found no significant differences in community structures between IBS and healthy controls at the amplicon sequence variants (ASV) level, machine learning (ML) approaches enabled us to discriminate IBS from healthy controls at genus level. Linear discriminant analysis effect size (LEfSe) analysis was subsequently used to seek out 97 biomarkers across all studies. Then, we quantified the standardized mean difference (SMDs) for all significant genera identified by LEfSe and ML approaches. Pooled results showed that the SMDs of nine genera had statistical significance, in which the abundance of *Lachnoclostridium, Dorea, Erysipelatoclostridium, Prevotella 9*, and *Clostridium sensu stricto 1* in IBS were higher, while the dominant abundance genera of healthy controls were Ruminococcaceae *UCG-005*, *Holdemanella*, *Coprococcus 2*, and *Eubacterium coprostanoligenes group*. In summary, based on six published studies, this study identified nine new microbiome biomarkers of IBS, which might be a basis for understanding the key gut microbes associated with IBS, and could be used as potential targets for microbiome-based diagnostics and therapeutics.

## Introduction

Irritable bowel syndrome (IBS) is a common gastrointestinal disorder characterized by chronic, recurrent episodes of abdominal discomfort and pain with altered bowel habits. It has affected approximately 12% of the global population and over 16% of the United States population (Schmulson et al., 2006; Poulsen et al., 2017; Ma et al., 2020). According to defecation pattern, IBS patients can be divided into four main subtypes, including IBS with constipation (IBS-C), IBS with diarrhea (IBS-D), IBS with mixed bowel habits (IBS-M), and unclassified IBS. Currently, the Rome criteria (Rome IV) has defined IBS as recurrent abdominal pain with at least one day of abdominal pain per week for the past three months and two or more of the following symptoms: pain related to defecation, change in fecal frequency, change in fecal shape, and any of these not less than six months before diagnosis (Hellstrom and Benno, 2019; Asghar et al., 2020; Palsson et al., 2020). IBS has become a significant disease burden in terms of increased absenteeism from school or work and reduced health-related quality of life, although it is not a fatal disease (Zhen Lu et al., 2006; Ma et al., 2020; Yao et al., 2020). IBS is a complex and heterogeneous disease with many factors involved in its etiology and pathogenesis (i.e., mucosal immune hyperactivity, food intolerances, distortions in the gut microbiome) (Quigley, 2005; Mullin et al., 2014; Moloney et al., 2016; Gonzalez-Castro et al., 2017).

An increasing number of studies have reported a critical role for the gut microbiome in health and IBS, respectively (Kassinen et al., 2007; Krogius-Kurikka et al., 2009; Carroll et al., 2011; Rajilic-Stojanovic et al., 2011; Jeffery et al., 2012), but trends in the microbial biomarkers associated with IBS in these reports are inconsistent. At the phylum level, some studies showed higher abundance of Proteobacteria in IBS (Carroll et al., 2012; Pozuelo et al., 2015), whereas other research showed no difference of Proteobacteria relative to healthy ones (Chung et al., 2016; Dior et al., 2016). At the genus level, *Faecalibacterium*, *Blautia* (Pozuelo et al., 2015; Tap et al., 2017), *Veillonella* (Tana et al., 2010; Rigsbee et al., 2012), and *Ruminococcus* (Pozuelo et al., 2015) were identified to be positively associated with IBS and *Methanogens* (Rajilic-Stojanovic et al., 2011) was depletion in IBS. A study indicated that *Lachnoclostridium* was significantly associated with the clinical symptoms of IBS (Zhu et al., 2019), while there was little concern in other studies. Thus, it remains uncertain whether there are highly reproducible microbial signatures that can differentiate IBS subjects from healthy controls across cohorts and study designs.

Meta-analysis has been used as a tool to establish and validate associations between the intestinal microbiome and diseases across populations or cohorts (Walters et al., 2014; Sze and Schloss, 2016; Duvallet et al., 2017; Sze and Schloss, 2018; Armour et al., 2019). Several meta-analyses have been performed based on studies linking the gut microbiome to IBS using bacterial culture and qPCR, or 16S rRNA gene technology (Liu et al., 2017; Zhuang et al., 2017; Wang et al., 2020). Nevertheless, these existing meta-analysis studies still have limitations. The bacteria cultivation and qPCR technologies (Kassinen et al., 2007; Krogius-Kurikka et al., 2009; Rajilic-Stojanovic et al., 2011) focused on only a limited number of microorganism species (Gerritzen et al., 2011). Analysis based on this evidence can only evaluate whether there are significant changes within “common” bacteria when IBS occurs, rather than looking for key bacterial characteristics without preconceived assumptions. For example, these articles highlighted only lower *Lactobacillus* and *Bifidobacterium* and higher *Escherichia coli* levels in IBS compared to the healthy group. This indicates that research based on non-targeted microbial identification methods, such as 16S rRNA gene technology, deserve more attention in meta-analysis. Besides, previous meta-analyses were almost always based on the final results of independent studies, rather than the raw sequencing data, which could not alleviate the incompatibility among multi-cohort efforts due to the variation in bioinformatics pipelines (Pollock et al., 2018). Meta-analyses based on raw sequencing data have successfully characterized the microbiome signatures in colorectal cancer, tumors (Shah et al., 2018; Sze and Schloss, 2018; Thomas et al., 2019), and obesity (Finucane et al., 2014; Walters et al., 2014; Sze and Schloss, 2016). Therefore, it is thus necessary to conduct such analyses to identify significant differences in the gut microbiome between IBS and healthy controls, which have not been reported to date.

Here, we collected 16S rRNA gene sequence data of stool samples (n=439) from six studies (Saulnier et al., 2011; Pozuelo et al., 2015; Labus et al., 2017; Zhuang et al., 2018; Lo Presti et al., 2019; Zhu et al., 2019). A unified pipeline was used to process raw sequencing data to investigate whether biomarkers describing bacterial communities or community-specific microbiota profiles could more accurately identify IBS and healthy controls. The results of our study showed that alterations in bacterial communities are indeed associated with IBS and that a subset of the bacterial profiles may be considered as potential biomarkers for identifying the presence of IBS.

## Materials and Methods

### Data Sets Collection

We followed previously published methods for the meta-analyses of microbiome data (Duvallet et al., 2017; Sze and Schloss, 2018). Raw data and metadata for the included cohorts were downloaded from the Sequence Read Archive (SRA). We collected 16S rRNA gene sequence data of 439 stool samples from six previously published studies, whose sequencing methods were performed using Illumina sequencing or 454 sequencing. We excluded studies that were reviewed or meta-analyzed, focused on cultivation and qPCR techniques, or were used only as abstracts for conference papers. Studies without controls or with fewer than five case patients were also excluded. Any studies that failed to provide either publicly available sequences or metadata were excluded. The reuse of these published data in our meta-analysis complied with all relevant ethical regulations. Of the included studies, two were from the US, two were from China, one was from Italy, and one was from Spain. We manually curated metadata tables for the public case-controls.

### Data Processing

Raw sequence data and metadata were obtained from the Sequence Read Archive (SRA) in NCBI. To avoid bias caused by different bioinformatic analysis pipelines, the sequence read pools for each study were filtered and analyzed using the same custom script based on the QIIME2-2020.2 (Hall and Beiko, 2018; Bolyen et al., 2019). Each dataset was imported and assembled in QIIME2-2020.2 against single-end sequences for 454 sequencing or paired-end sequences for Illumina-sequencing. Denoising was implemented using DADA2 (divisive amplicon denoising algorithm 2) which discards chimeras and erroneous sequences (Callahan et al., 2016). The individual real biological sequences, referred to as amplicon sequence variants (ASV) (Callahan et al., 2017) were retrieved as a higher resolution version of the operational taxonomic unit (OTU) table than those generated by traditional methods, as well as their frequencies. All remained sequences had a length of ≥200 bp and an average sequence quality score of ≥20. Subsequently, the resulting representative set of sequences was aligned and classified using the SILVA database (Quast et al., 2013; Yilmaz et al., 2014). ASVs with fewer than 10 reads were removed. We normalized the relative abundance of each ASV by dividing its value by the total number of reads for each sample. We then collapsed the ASVs to the genus level by summing their respective relative abundances, discarding any ASVs which were unannotated at the genus level. All statistical analyses were performed on the relative abundance data at this genus level.

### Community Analysis

Both alpha and beta diversity analyses were performed within each dataset through the q2-diversity plugin in QIIME2. Alpha diversity metrics (i.e., evenness, observed-OTUs, and Shannon) were calculated based on ASV level. The non-parametric Kruskal-Wallis test (Sculco, 2001; Ren et al., 2018) was used to test alpha diversity metric dissimilarities, and the p-value was corrected using the Benjamini-Hochberg method (Ferreira, 2007; Abbas et al., 2013). The Bray-Curtis distance was used to measure beta diversity metrics. We explored the community structure of the samples with PERMANOVA (Kelly et al., 2015) using the beta-group-significance command. Rarefaction was performed on the feature table before calculating the distances in QIIME2.

### Statistical Analysis

Two classification algorithms were used to classify healthy and IBS individuals. We built an AdaBoost classifiers (Montazeri et al., 2016) function with 1000 estimators utilizing Python’s scikit-learn module (Pedregosa et al., 2011), and random forest classification models using the RandomForest package (Lebanov et al., 2020). All models were built with 10-fold cross-validation using data at the genus level. Based on the test results of cross-validation, the interpolated area under the receiver operating characteristic (ROC) curve (AUC) was calculated. AUC represents the area under the ROC curve, which can evaluate the classification ability of machine learning models. The higher the AUC, the better the model at correctly classifying instances, and 0.5 is the decision threshold.

**Figure 1:**
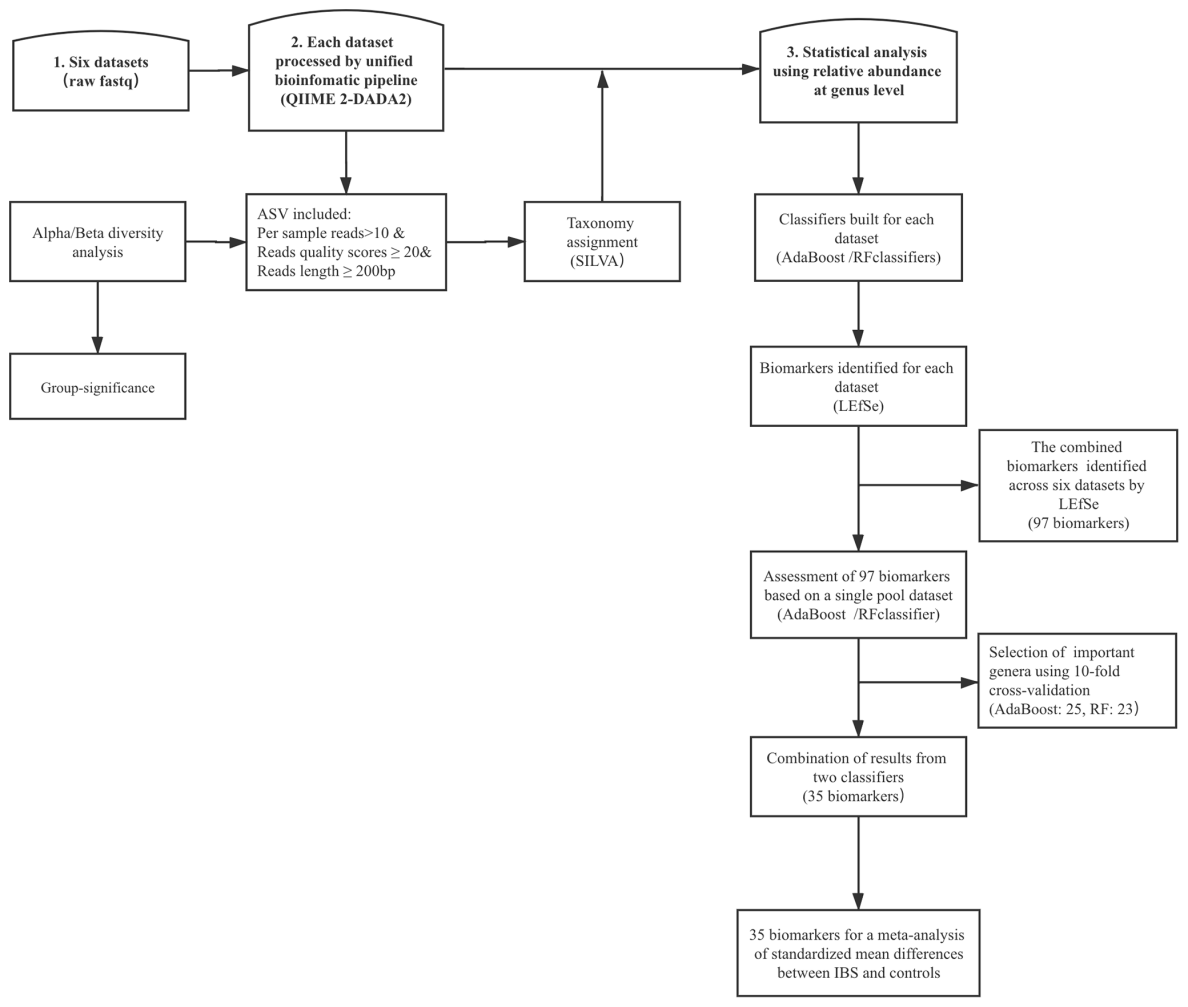
The top frames represent the three main steps, and the rectangular frame shows the data process. DADA2, divisive amplicon denoising algorithm 2; ASV, amplicon sequence variants; LEfSe, linear discriminant analysis effect size; RF, random forest; IBS, irritable bowel syndrome.

Univariate analysis based on the relative abundances of genera was performed using the linear discriminant analysis effect size (LEfSe) method (Segata et al., 2011). We focused on those biomarkers that had significant difference between cases and controls per dataset, and then combined these results across all studies. Finally, we performed fixed-effects model based on all important features and estimated the effect size standardized mean differences (SMDs). The entire analytical workflow is shown in **Figure 1**.

## Results

A total of 106 studies were identified in PubMed (**Figure S1**). A total of 31 studies that were reviews or animal studies were excluded. Of the 75 full-text articles reviewed for eligibility, 52 studies that did not meet certain eligibility requirements were discarded, and 23 studies had datasets with inclusion criteria. However, 17 datasets were excluded due to unavailable raw data or incomplete metadata. We finally collected and re-processed the original stool 16S rRNA sequence data published in six studies, including 253 IBS samples and 186 control samples (Saulnier et al., 2011; Pozuelo et al., 2015; Labus et al., 2017; Zhuang et al., 2018; Lo Presti et al., 2019; Zhu et al., 2019). A summary of the included studies, data characteristics, and metadata is presented in **Table 1**.

**Table 1:** Size and characteristics of the IBS 16S rRNA datasets included in this study.

Dataset	Groups (n)	Age (average ± sd.)	BMI (average ± sd.)	Sex F/M	Country	Data storage (NCBI SRA)	Amplification region	Sequencing method
Zhuang_2018 (Zhuang et al., 2018)	HC (13)	30.54 ± 6.75	20.84 ± 1.46 20.61	8/5 21/9	China	SRP150089	V3–V4	Illumina-MiSeq
	IBS-D (30)	32.1 ± 8.11	± 3.26					
Presti_2019 (Lo Presti et al., 2019)	HC (47), IBS (44)	54, 48	23, 24	20/27, 30/14				
	IBS-D (16)							
	IBS-C (18)	NA	NA	NA	Italy	SRP110018	V1-V3	454 GS Junior
	IBS- A (10)							
Pozuelo_2015 (Pozuelo et al., 2015)	HC (66), IBS (113)	37.6 ± 13, 42.6 ± 13	23.7 ± 3.4, 23.7 ± 4	40/26, 80/33				
	IBS-D (54)	41.9 ± 13	25 ± 4.6	29/25				
	IBS-C (32)	39.4 ± 10.8	23.3 ± 3.8	31/2	Spain	SRP050404	V4	Illumina-MiSeq
	IBS-M (27)	48.2 ± 16.4	23.9 ± 3.6	32/8				
Zhu_2019 (Zhu et al., 2019)	HC (15)	28.27 ± 1.56	NA	7/8	China	SRP222428	V4	Illumina-HiSeq
	IBS(15)	47.67 ± 14.24		1/2				
Saulnier_2011 (Saulnier et al., 2011)	HC (22), IBS (22)	9.32 ± 1.52, 9.41 ± 1.04	NA	11/11, 8/14	US	SRP002457		454 GS FLX
	IBS-D (1)	9.38 ± 1.19		5/8				
	IBS-C (13)	10		0/1			V1-V3	
	IBS-U (7)	9.26 ± 1.89		3/4			V3-v5	
	other (1)	9		0/1				
Labus_2017 (Labus et al., 2017)	HC (23), IBS (29)	26.0 ± 6.48, 26.1 ± 5.72		14/9, 21/8				
	IBS-D (10)							
	IBS-C (11)							
	IBS-M (5)	NA	NA	NA	US	SRP099239	V3-V5	454 GS FLX
	IBS-A (1)							
	IBS-U (2)							

NA, data not available; IBS, irritable bowel syndrome; HC, healthy controls; IBS-D, diarrhea-predominant IBS; IBS-C, constipation-predominant IBS; IBS-M, mixed IBS; IBS-A, alternating IBS; IBS-U, unsubtyped IBS.

### No Consistent Significant Shifts in Community Diversity Analysis

We firstly assessed whether there was a divergence in microbial community composition and distribution at the ASV level between healthy controls and IBS patients. For the alpha diversity metrics, evenness, observed-OTUs, and Shannon were calculated. The result of the K-W test showed that only two of the six studies had statistically significant differences between the two groups (Pozuelo et al., 2015; Zhu et al., 2019), according to the Shannon and observed-OTUs indices (**Table S1**). To test for differences in microbiota profiles between IBS disease stages and healthy controls, we performed PERMANOVA analysis within each dataset based on the Bray-Curtis distance (**Table S2**). The results demonstrated that the statistically different studies were consistent with those found in alpha diversity analysis (Pozuelo et al., 2015; Zhu et al., 2019), and a study had a p-value marginally greater than 0.05 (Saulnier et al., 2011). These results implied that from a microbial perspective, the correlates of IBS might be some of the key taxa found, rather than the the entire community structure depicted at the level of ASVs or OTUs.

### Machine Learning Algorithm Showed Microbiological Changes at the Genus Level

**Figure 2:**
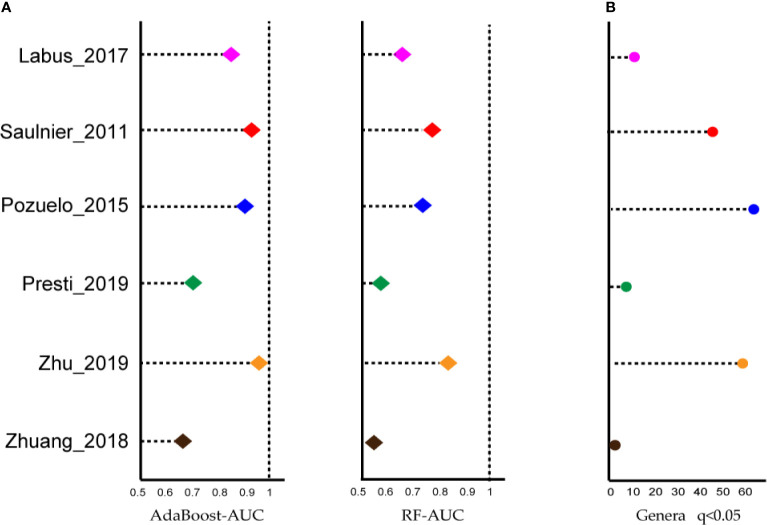
Most of the studies showed microbiome changes, and potential signatures were found at genus-level in each dataset. **(A)** Left: Area under the ROC curve (AUC) is calculated by the AdaBoost algorithm. Right: Area under the ROC curve (AUC) is calculated by the random forest algorithm. X-axis starts at 0.5. **(B)** The number of genera biomarkers with p < 0.05 were identified by linear discriminant analysis effect size (LEfSe) analysis.

Next, we wondered whether the relationship between intestinal microbiota and IBS would be recapitulated when rearranging microbiological data at a higher taxonomic level. To utilize the biodiversity data more effectively, two sensitive ML algorithms, AdaBoost and random forest classifiers, were employed at the genus level, to estimate whether the gut microbiota was altered in IBS, and to distinguish cases from controls, respectively. As shown in **Figure 2A**, both classifiers, especially the AdaBoost, provided AUC values greater than 0.8 in at least four datasets. These results indicated that although the ASV results shown above were not expected, IBS patients carry an altered gut microbiota that can be correctly classified by genus-level gut microbiome data using the ML method.

### Identified Significant Microbiome Biomarkers

The linear discriminant analysis effect size (LEfSe) tool was utilized to search for bacterial biomarkers within each dataset. After pooling respective biomarkers from six datasets, a total of 97 biomarkers (LDA SCORE > 2) were identified (**Figure 2B**). However, the great majority of genera were dataset-specific, with only *Parabacteroides* genera being shared in four of the six studies and four genera being identified in 3/6 studies, i.e., *Lachnospiraceae_NK4A136_group*, *Butyricimonas*, *Dorea*, and *Lachnoclostridium*.

**Figure 3:**
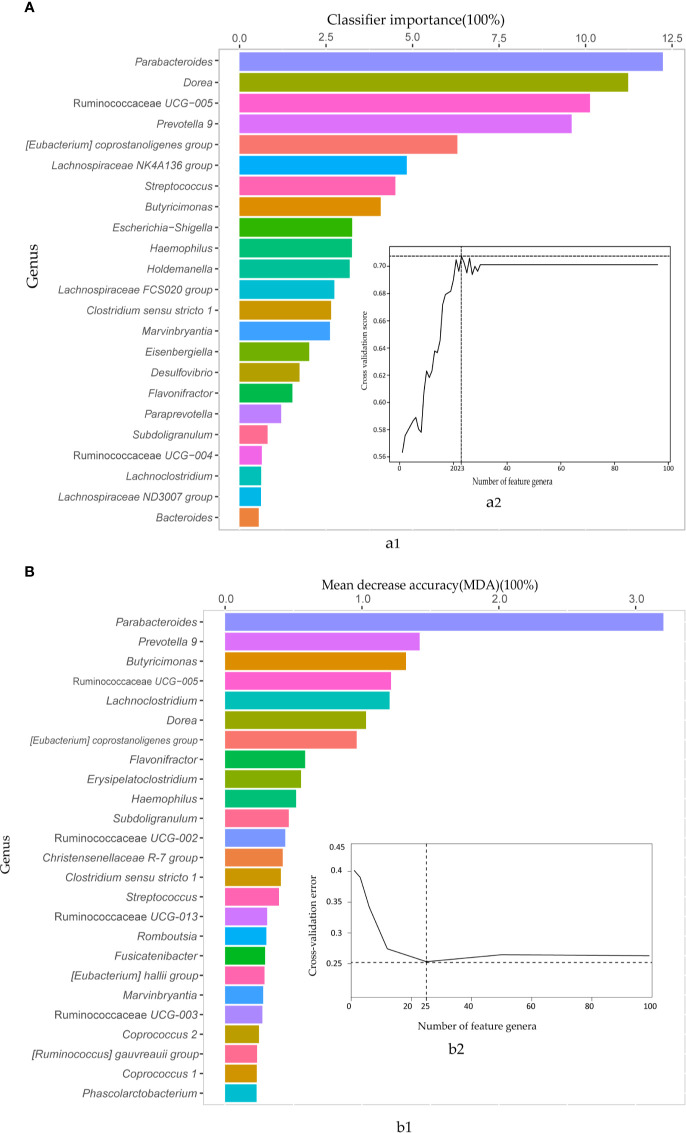
The AdaBoost and random forest classifiers identified important feature genera in distinguishing IBS from healthy subjects. **(A)** The important features selected by the AdaBoost classifier. a1. The top 23 genus biomarkers were ranked in descending order of the most relevant features to the model. a2. 10-fold cross-validation score on the Y-axis and the number of features on the X-axis. **(B)** The important features contributed to random forest corresponding to AdaBoost. b1. 25 important genera sorted in descending order based on mean decrease accuracy (MDA). b2. 10-fold cross-validation error on the Y-axis and the number of features on the X-axis.

Then, the AdaBoost and random forest classifiers were built based on the 97 biomarkers to further assess the combined biomarkers selected by LEfSE analysis. We observed an impressive classification with an AUC value of 0.86 (random forest) and 0.77 (AdaBoost), separately (**Figures S2, S3**). To obtain the essential taxa, after performing feature selection and pruning random forest trees, the AdaBoost and random forest classifiers retained the 23 and 25 most important features, respectively (**Figure 3**). The most crucial genus was *Parabacteroides*, and other features included genera such as *Dorea*, Ruminococcaceae *UCG-005*, and *Prevotella 9*. Thus, to avoid the loss of essential genera information, we pooled the results from the two classifiers, and got 35 important features.

**Figure 4:**
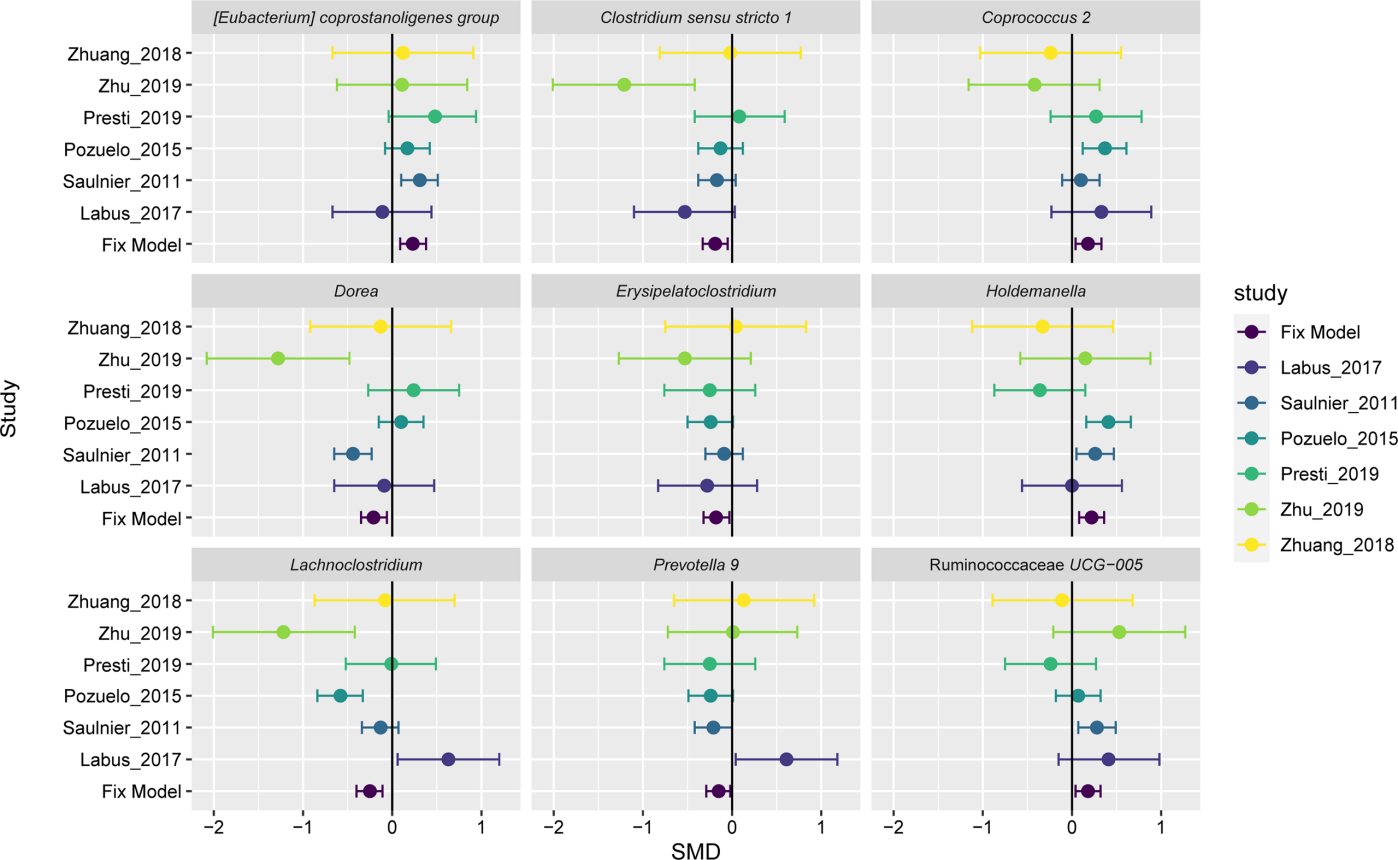
Forest plot reporting effect sizes calculated using a meta-analysis of standardized mean differences and a fixed-effects model on nine genera abundances between carcinomas and controls. The SMD had a positive value confirming the higher number of genera in patients with IBS, and a negative value showing more abundance of genera in healthy controls. The length of the error bar depicts the 95% CIs. The fixed model indicates the overall effect sizes SMD value of all studies.

A fixed-effect model was used to aggregate these pieces of evidence in order to accurately assess the degree of consistency of bacterial biomarkers of IBS disease across datasets. Of the 35 important genera mentioned above, we found nine genera had statistically significant difference between the two groups across studies (**Figure 4**). Three of these significant genera showed a significant decrease in the impurity of the random forest tree, such as *Erysipelatoclostridium* (SMD=-0.18, 95% CI: -0.32 to -0.03; P= 0.0150), *Clostridium sensu stricto 1* (SMD=-0.19, 95% CI: -0.33 to -0.05; P= 0.0091), and *Coprococcus 2* (SMD=0.18, 95% CI 0.04 to 0.32; P= 0.014). *Holdemanella* (SMD=0.22, 95% CI 0.08 to 0.36; P = 0.0025) contributed only to the AdaBoost classifier. And five genera were ranked as highly important in both AdaBoost and random forest classifiers: the genera *Dorea* (SMD=-0.21, 95% CI: -0.35 to -0.06; P= 0.0050), *Prevotella 9* (SMD=-0.15, 95% CI: -0.30 to -0.01; P= 0.0399), *Lachnoclostridium* (SMD=-0.25, 95% CI: -0.40 to -0.11; P= 0.0006), Ruminococcaceae *UCG-005* (SMD=0.18, 95% CI 0.04 to 0.32; P= 0.0140), and *Eubacterium coprostanoligenes group* (SMD=0.23, 95% CI: 0.09 to 0.38; P= 0.0013).

The results of the overall analysis provide robust evidence for a quantitative genus distribution of microorganisms associated with IBS, such as the nine microbiome biomarkers. The genera *Erysipelatoclostridium*, *Clostridium sensu stricto 1*, *Dorea*, *Prevotella 9*, and *Lachnoclostridium* were significantly elevated in IBS patients relative to controls, while *Coprococcus 2, Holdemanella*, Ruminococcaceae *UCG-005*, and *Eubacterium coprostanoligenes group* had a richer abundant in the controls compared to the IBS group. The variation trend for three genera, *Dorea* (Saulnier et al., 2011), *Lachnoclostridium*, and *Clostridium sensu stricto 1* (Zhu et al., 2019), were largely consistent with the initially reported results. The other statistically significant genera have hardly been highlighted in previous IBS microbiome studies or meta-analyses, and their biological functions related to IBS have not been reported so far. In despite of the inevitable heterogeneity, the nine genera signatures were important in classification. Therefore, our findings on repeatable microbial signatures for IBS might be helpful to design non-invasive diagnostic tools.

## Discussion

Previous studies on dysbiosis of the gut microbiome in IBS have been reported (Zhuang et al., 2017; Pittayanon et al., 2019; Wang et al., 2020). However, our research is the first meta-analysis based on raw sequencing data with the aid of two ML procedures. By collecting data from stool samples across studies, and analyzing them in a unified pipeline, we reassessed the role of the microbiome in IBS, and investigated several novel microbiota bacteria at the genus level that have been barely highlighted in previous studies. Importantly, our findings consolidate and extend previous reports that the gut microbiota was significantly associated with IBS and may provide a potential tool for non-invasive diagnosis. The development of non-invasive microbial biomarkers will make it possible for the diagnosis and guidance of drug use (Yu et al., 2017; Liang et al., 2020). For example, patients who are characterized by depletion of health-associated microbes could take treatments of probiotics or other interventions to enrich these taxa. Studies committed to colorectal cancer have taken a step further towards non-invasive microbial biomarkers in fecal samples. Therefore, it will be beneficial to excavate IBS-related microbial non-invasive tools.

The disease tends to have an alterated microbiome profile. However, we observed inconsistent results of community diversity analysis between previous studies. Some studies report no significant difference in Shannon diversity (Labus et al., 2017; Tap et al., 2017; Jeffery et al., 2020), and others show reduced microbiota richness in the IBS group (Durban et al., 2012; Lo Presti et al., 2019). A recent meta-analysis from Lin also had an uncertain result for changes in α-diversity (Wang et al., 2020), because the included studies reported conflicting findings, or too few studies were available. Fortunately, although our results also showed that the significance of α-diversity or β-diversity differences is not a universal phenomenon, the two ML classifiers based on global genus-level data can comprehensibly classify IBS disease from healthy status. It implies that the ML algorithm might play a more significant role in the discovery of microbial profile differences.

Actually, the approach based on the machine learning algorithm has been involved in multiple medical fields, such as some case-control and cohort studies (Koyner et al., 2018; Kanda et al., 2019; Pasolli et al., 2019; Inaguma et al., 2020). Compared to classic approaches, ML procedures allow researchers to reduce the complexity of extensive data into specific classifications, shorten the computational cost time, and improve accuracy. A previous study reported no differences in fecal microbiota abundance or composition between healthy and IBS groups through classic ecologic approaches, but identified a microbial signature for IBS severity while using ML (Tap et al., 2017). Furthermore, although the results of the two algorithms differed, the use of complementary variables for the analysis allowed us to take full advantage of each analytical technique to provide more comprehensive and accurate information (Miller and Moore, 2014; Stadler and Mukherjee, 2017). There are also some inevitable limitations of ML models (Brynjolfsson and Mitchell, 2017). For example, when they do make errors, diagnosing and correcting them can be difficult because it will require going through the underlying complexities of the algorithms and associated processes; the bigger the data, the longer it will take. However, ML has been evolving, and our evidence confirms that this combined approach should be credible and reliable in the research of microbial markers.

Our study suggested that biomarkers selected by LEfSE could correctly classify IBS and healthy controls, and then nine key signatures were screened by ML algorithm and meta-analysis. The biological functions of the species belonging to them have been previously indicated. For instance, the *Prevotella* strains are known to be associated with chronic inflammatory conditions (Ley, 2016; Larsen, 2017). The species *Holdemanella biformis* acting as a histone deacetylaseinhibitor (HDACi) affects the activation of calcineurin and nuclear factor of activated T cells (NFAT)c3, which leads to the inhibition of tumor cell growth (Zagato et al., 2020). Both the presence of inflammation and the increased mast cell density play a critical role in the disease process of IBS, and has clinical significance (Ng et al., 2018). Besides, future studies should be designed to explore the exact biological function of these taxa, and to understand any potential role of these genera in the progression of IBS.

The genus *Parabacteroides* showed the largest contribution in the two classifiers. Previous authors also argued that its abundance correlated with 15Phe allele dosage in the sucrase–isomaltase gene, which is strongly associated with increased risk of IBS (Rangel et al., 2015). Nevertheless, there was no significant difference between two groups by meta-analysis across six studies. This finding should be viewed in the context of considerable variations in clinical and individual characteristics of included studies. For example, the researchers found differences between obese and lean individuals in this genus (Wu et al., 2019). This again hints that the ML algorithm plays a good role in disease-related microbiome analysis.

Another interesting discovery is that the genus *Brachyspira* (yet not important for any classifiers) was only present in the pediatric dataset included in our analysis. It was observed in patients with IBS that *Brachyspira* attached to the colonocyte apical membrane and linked with mast cell activation, mild mucosal inflammation, and changes of molecular pathways related to bacterial uptake (Jabbar et al., 2020). However, *Brachyspira* was not identified in the original study (Saulnier et al., 2011), possibly due to the different representative sequences or an outdated reference database (Quast et al., 2013; Duvallet et al., 2017; Mancabelli et al., 2017; Gibbons et al., 2018; Armour et al., 2019). In this meta-analysis, the generation of representative sequences was based on ASVs, rather than OTUs. In general, ASVs have inherent biological significance to the DNA sequence, and provide more comprehensive inference from large marker-gene datasets, relative to *de novo* OTUs. Existing ASVs provide better sensitivity and precision than the OTU methods (Callahan et al., 2017; Caruso et al., 2019). Consequently, further applications of ASV methods and database updates should be recommended.

We adhered to a uniform pipeline for conducting the meta-analysis. However, heterogeneities are common and inevitable in meta-analyses. This was also observed in three other meta-analyses that including larger studies than ours. The inconsistency in the microbial profiles in the stools may be due to complicated and various factors among studies, for instance, individual experimental design (Sample storage, DNA extraction technique, 16S rRNA target region) (Lozupone et al., 2013; Mancabelli et al., 2017), host-related covariates (diet, antibiotics, inflammation, age, geography, temporal, culture, and race) (Wu et al., 2011; Yatsunenko et al., 2012; Gorvitovskaia et al., 2016; Staudacher and Whelan, 2016; Rej et al., 2018; Zhong et al., 2019), and disease assessment. *Erysipelatoclostridium* has been reported to be positively associated with dairy intake (Shen et al., 2019). Abundance of *Holdemanella* has been observed to correlate with gender (Min et al., 2019). Therefore, the availability of comprehensive metadata might help to stratify the analysis and attenuate heterogeneity in further meta-analyses. Unfortunately, we failed to assess how our microbial findings were associated with the IBS subtype due to the lack of sufficient metadata. To make better use of the data collected in the original studies, data were unified and pooled to analyze instead of being segregated by subgroup to gain greater power. Meanwhile, numerous studies will need to be included to lessen substantial heterogeneities for the meta-analysis. And we highlight the significance of making raw data and associated patient metadata publicly available to enable more comprehensive analyses in the future.

Our meta-analysis with its deficiency adds new knowledge that the nine genera play an important role in distinguishing healthy individuals from the IBS group. Besides, we built ML classification models using these nine biomarkers. The AUC value of RF was 0.77, but the AdaBoost classifier had an AUC value about 0.6. This phenomenon might be caused by heterogeneity and other confounding factors. For instance, IBS is a complex and heterogeneous disease with many factors involved in its etiology and pathogenesis. Despite the unfortunate performance of these nine genera, our results provide new insight into the microbiome dysbiosis of IBS. Further studies require targeted functional analysis of these nine signatures in IBS. Besides, we demonstrate that meta-analysis combined with machine learning algorithms may be a responsible approach in microbiome and disease research. Furthermore, meta-analysis can allow for further stratification of disease subtypes and microbiome disruption if potential influencing factors such as unavailable raw sequencing data or metadata, ambiguous and incomplete metadata can be addressed. In summary, this work demonstrates the feasibility of using a unified bioinformatics approach to pursue new findings in the broader field of clinically relevant microbiome research and enhances the value of separate analyses. As the field evolves, researchers should utilize an increasing number of replicated case-control studies to effectively translate putative microbiome ideals into clinical practice.

## Data Availability Statement

Publicly available datasets were analyzed in this study. This data can be found here: NCBI, accession numbers: SRP002457, PRJNA566284, SRP150089, PRJNA391149, PRJNA268708, PRJNA373876.

## Author Contributions

YL: writing-original draft preparation. WL, HY, XZ, and WW: methodology and visualization. LM, YW, and HZ: writing—review and editing. SJ, LC, and XT: supervision. LW, YJW, JS, and YS: project administration. All authors contributed to the article and approved the submitted version.

## Funding

This study was funded by the Science and Technology Key Program of Tianjin (No.19ZYPTJC00060).

## Conflict of Interest

Authors LW, YW, JS and YS were employed by company Tianjin Zhongxin Pharmaceutical Group Co., Ltd.

The reamining authors declare that the research was conducted in the absence of any commercial or financial relationships that could be construed as a potential conflict of interest.
