# Supplementary material for: Leveraging 16S rRNA Microbiome Sequencing Data to Identify Bacterial Signatures for Irritable Bowel Syndrome
Source: Front Cell Infect Microbiol. 2021 Jun 11;11:645951. doi: 10.3389/fcimb.2021.645951 (PMC8231010; doi:10.3389/fcimb.2021.645951)
Supplement: Supplementary file 1 [file DataSheet_1.zip › Supplementary Files/Figure-S1.pdf]

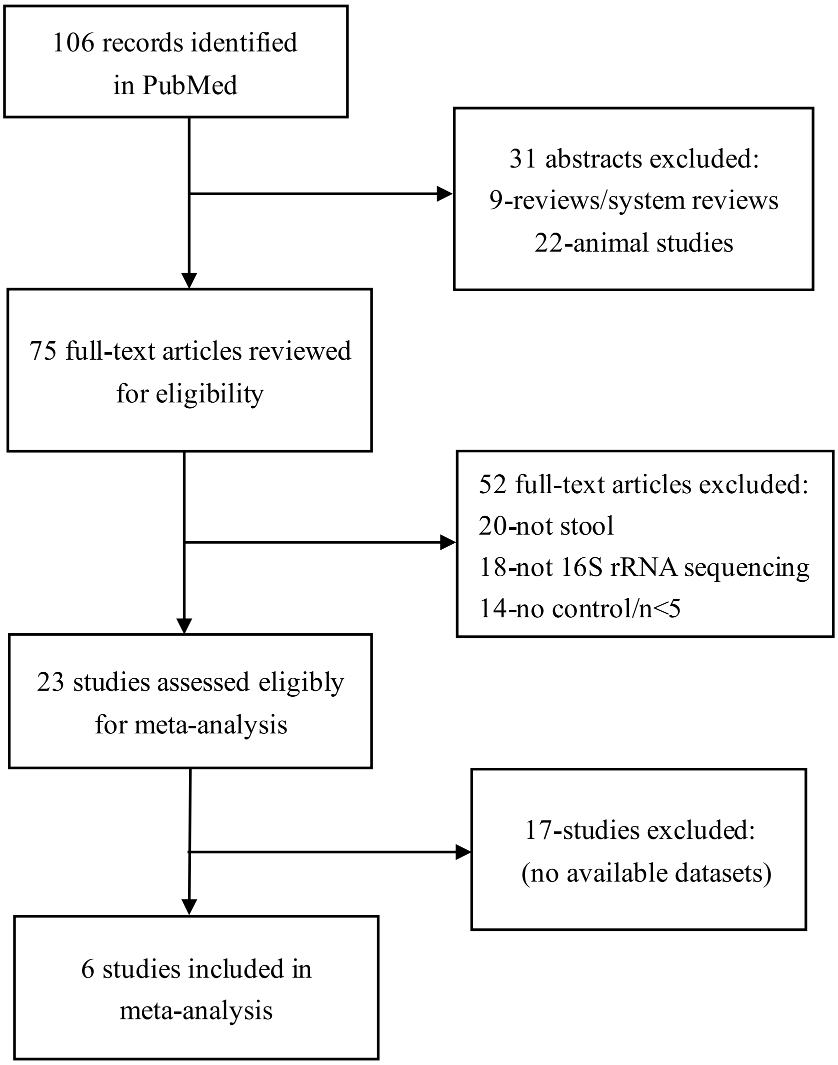

Figure. S1 The flow diagram depicts our search results and selection of included studies in this meta-analysis.
