# Supplementary material for: Leveraging 16S rRNA Microbiome Sequencing Data to Identify Bacterial Signatures for Irritable Bowel Syndrome
Source: Front Cell Infect Microbiol. 2021 Jun 11;11:645951. doi: 10.3389/fcimb.2021.645951 (PMC8231010; doi:10.3389/fcimb.2021.645951)
Supplement: Supplementary file 1 [file DataSheet_1.zip › Supplementary Files/Table-S2.pdf]

Table S2

Bray-curtis-distance-matrix		
Study	P-value	Q-value
Zhuang_2018	0.67	0.67
Zhu_2019	0.001	0.001 **
Presti_2019	0.331	0.343
Pozuelo_2015	0.001	0.001 **
Saulnier_2011	0.054	0.054
Labus_2017	0.946	0.946

*Q < 0.05 was considered statistically significant.
