# Supplementary figures and images for: Leveraging 16S rRNA Microbiome Sequencing Data to Identify Bacterial Signatures for Irritable Bowel Syndrome
Source: Front Cell Infect Microbiol. 2021 Jun 11;11:645951. doi: 10.3389/fcimb.2021.645951 (PMC8231010; doi:10.3389/fcimb.2021.645951)

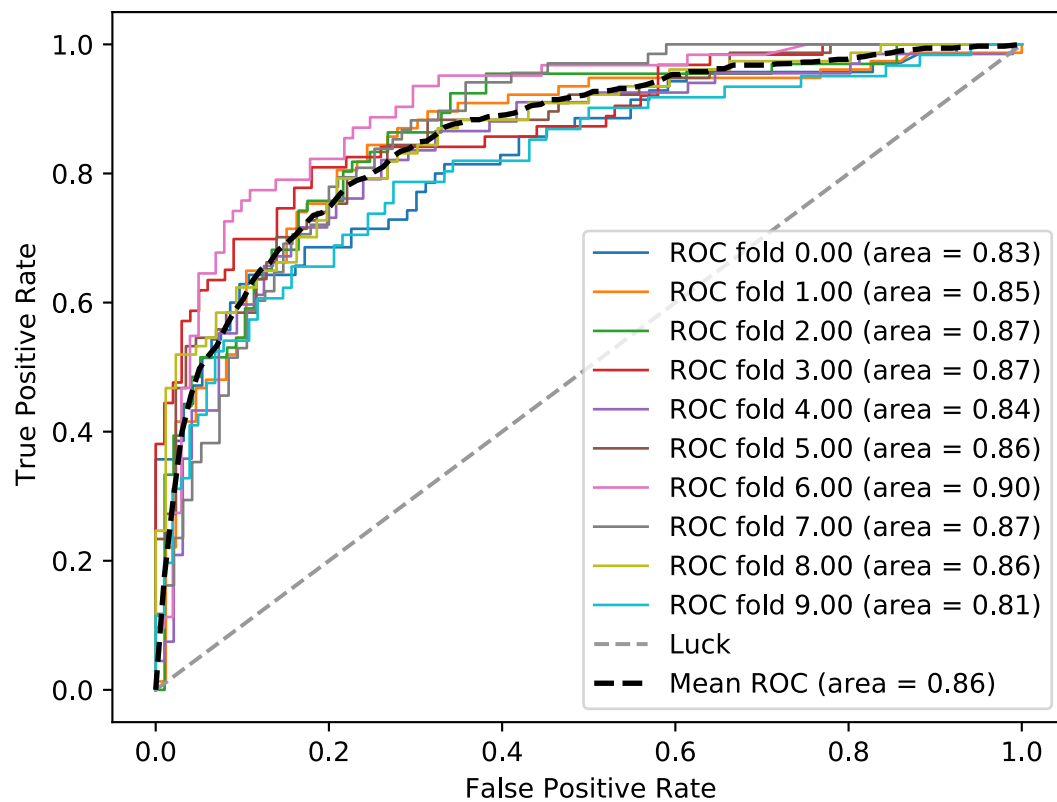

Supplement: Supplementary file 1 [file DataSheet_1.zip › Supplementary Files/Figure-S2.pdf]

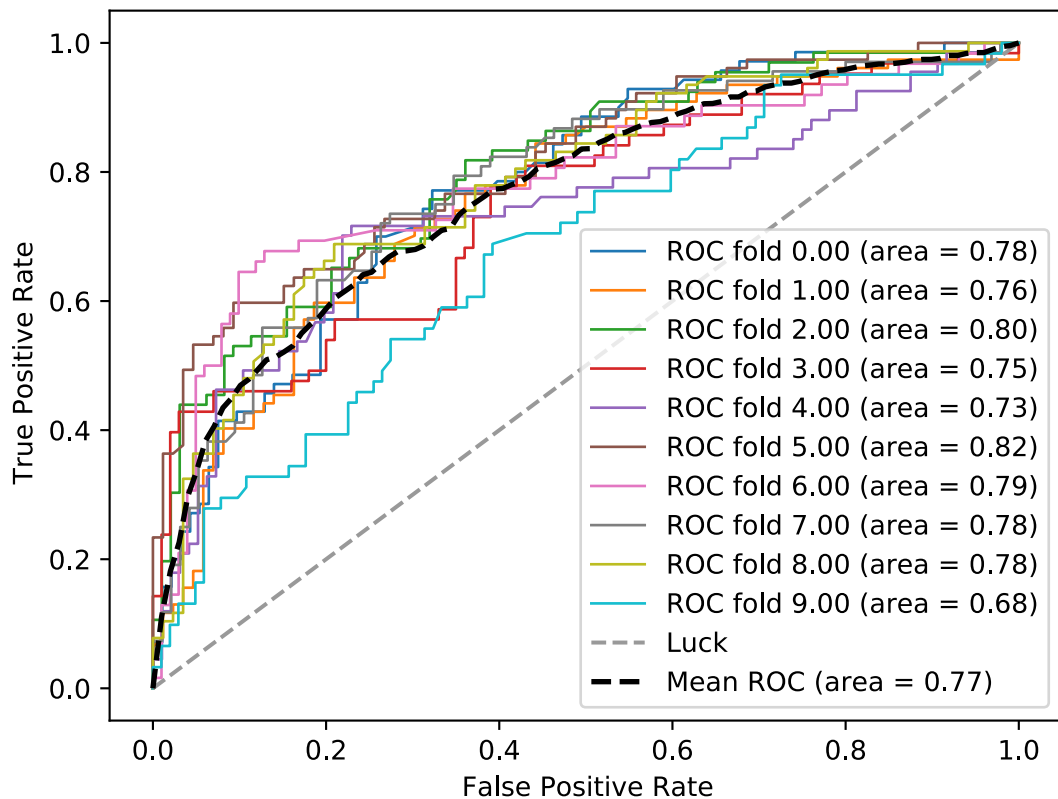

Supplement: Supplementary file 1 [file DataSheet_1.zip › Supplementary Files/Figure-S3.pdf]
